# Resting state brain networks in the prairie vole

**DOI:** 10.1038/s41598-017-17610-9

**Published:** 2018-01-19

**Authors:** Juan J. Ortiz, Wendy Portillo, Raul G. Paredes, Larry J. Young, Sarael Alcauter

**Affiliations:** 1 Instituto de Neurobiología, Universidad Nacional Autónoma de México. Boulevard Juriquilla 3001, Queretaro, 76230 Mexico; 2 Department of Psychiatry and Behavioral Sciences, Silvio O. Conte Center for Oxytocin and Social Cognition, Center for Translational Social Neuroscience, Yerkes National Primate Research Center, Emory University, 954 Gatewood Rd., Atlanta, GA 30322 USA

## Abstract

Resting state functional magnetic resonance imaging (rsfMRI) has shown the hierarchical organization of the human brain into large-scale complex networks, referred as resting state networks. This technique has turned into a promising translational research tool after the finding of similar resting state networks in non-human primates, rodents and other animal models of great value for neuroscience. Here, we demonstrate and characterize the presence of resting states networks in *Microtus ochrogaster*, the prairie vole, an extraordinary animal model to study complex human-like social behavior, with potential implications for the research of normal social development, addiction and neuropsychiatric disorders. Independent component analysis of rsfMRI data from isoflurane-anestethized prairie voles resulted in cortical and subcortical networks, including primary motor and sensory networks, but also included putative salience and default mode networks. We further discuss how future research could help to close the gap between the properties of the large scale functional organization and the underlying neurobiology of several aspects of social cognition. These results contribute to the evidence of preserved resting state brain networks across species and provide the foundations to explore the use of rsfMRI in the prairie vole for basic and translational research.

## Introduction

Resting state functional magnetic resonance imaging (rsfMRI) is a neuroimaging tool that allows the study of spontaneous brain functional organization. Specifically, when no task is imposed and even under light anesthesia, the low frequency fluctuations (<0.1 Hz) of the blood oxygen level dependent (BOLD) signal remain highly synchronized within the sensory, motor and associative networks in the human brain, defining the so called resting state networks^1–4^. This tool has proven its value to explore normal and altered brain function across the life span, especially in conditions where consistent stimulation or task performance is challenging, including early development^5–8^, aging^9,10^, neurological^11,12^ and psychiatric disorders^13,14^.

Several resting state networks have been consistently identified in rodents^15–23^, non-human primates^24–28^ and humans^1,2,4^ turning this technique into a promising tool for translational research. Among these networks, the default mode network^29–31^, mainly composed by the medial prefrontal, posterior cingulate and lateral parietal cortices, has been largely studied in animal models because alteration of its connectivity has been associated with a variety of neuropsychiatric disorders in humans^32–35^. For example, alterations of this network, using rsfMRI, have been observed in animal models of depression^36^, alzheimer^37^ and autism^38^, among others. These types of studies can help elucidate the potential underlying mechanisms of the corresponding human phenotypes.

The power of translational research however, relies on the use of appropriate animal models in order to emulate human conditions or pathology. In particular, the neurobiology of human-like social cognition has been difficult to study in typical laboratory animals, because they do not exhibit much of the complex social behavior observed in humans, including the formation of lasting social attachment. The prairie vole (*Microtus ochrogaster*) has emerged as a valuable animal model to study several aspects of social cognition, because they establish long-term socially monogamous relationships with mates, provide biparental care and in natural habitats, alloparental behavior is commonly observed^39,40^. This model has already contributed to better understand the neurobiology and genetics of social bonding^41–45^, parental care^46–48^, social buffering^49–51^, the effects of early life experience in later social behavior^52–54^ and social related depression^55–58^. A recent MRI study in the prairie vole demonstrated that unilateral whisker stimulation and the presentation of novel odors induces robust BOLD signal changes^59^, supporting further neuroimaging experiments. However, resting state networks have not been described in the prairie vole brain, which ultimately would expand the available tools for basic and translational research of this species.

Here, we explored the resting state functional connectivity in a group of prairie voles and hypothesized that large-scale brain networks would be evident, including primary sensory and associative networks like the default mode network, typically described in other rodents^15,19,22^, ferrets^60^, non-human primates^24,25,28^ and humans^29–31^. With this aim, we applied independent component analysis, a data-driven method that identifies independent connectivity patterns, on rsfMRI datasets of anesthetized prairie voles. Our findings confirm the presence of consistent resting state networks, strongly supporting the use of neuroimaging to characterize the functional organization of the prairie vole brain, with potential applications in the basic and translational research of social-related behaviors.

## Methods

### Animals

Thirteen male prairie voles (*Microtus ochrogaster*), 12 weeks old (54.15 ± 5.24 g), were studied. All animals were housed in a room with controlled light (14:10 light-dark cycle) and temperature 23 °C. Subjects were provided with rabbit diet HF-5326 (LabDiet, St. Louis, MO, USA), oat, sun flower seeds and water *ad libitum*. Animals were descendants of 6 mating pairs generously donated by Dr. Larry J. Young from his colony at Emory University. All animals were anesthetized with isoflurane in an air mixture at 3% concentration for induction and positioning at the scanner, and 1% to maintain sedation level during image acquisition. The head was immobilized using a bite bar and the coil head holder. Body temperature was monitored using a rectal probe (SA Instruments, Inc. Stony Brook NY) and maintained with the aid of circulating warm water within the animal holder. Respiration rate was monitored with an MR-compatible pneumatic pillow sensor (SA instruments Inc, Stony Brook NY). After the MR scanning session, animals fully recovered and were transferred back to their housing. All procedures were carried out in accordance with the “Reglamento de la Ley General de Salud en Materia de Investigación para la Salud” of the Mexican Health Ministry which follows the National Institutes of Health’s “Guide for the Care and Use of Laboratory Animals” (NIH Publications No. 8023, revised 1978). Animal research protocols were approved by the Instituto de Neurobiología animal care committee.

### Image acquisition

MRI acquisition was conducted with a Bruker Pharmascan 70/16US, 7 Tesla MR scanner (Bruker, Ettlingen, Germany), using an MRI CryoProbe transmit/receive surface coil (Bruker, Ettlingen, Germany). Imaging protocols were performed using Paravision-6 (Bruker, Ettlingen, Germany). First, an anatomical scan was acquired using a spin-echo rapid acquisition with refocused echoes (Turbo-RARE) sequence with the following parameters: repetition time (TR) = 1800 ms, echo time (TE) = 38 ms, RARE factor = 16, number of averages (NA) = 2, field of view (FOV) = 18 × 20 mm^2^, matrix dimension (MD) = 144 × 160, slice thickness = 0.125 mm, resulting in isometric voxels of size 0.125 × 0.125 × 0.125 mm^3^. Before running the fMRI sequence, local field homogeneity was optimized within an ellipsoid covering the skull using previously acquired field maps. BOLD rsfMRI was acquired using a 10 minute long spin-echo echo planar imaging (SE-EPI) sequence: TR = 2000 ms, TE = 19 ms, flip angle (FA) = 90°, FOV = 18 × 16 mm^2^, MD = 108 × 96, yielding an in-plane voxel dimension of 0.167 × 0.167 mm^2^, and slice thickness of 0.7 mm,

### Brain template construction

Anatomical scans of seven voles were used to create a prairie vole brain template. First, images were bias field corrected using N4ITK^61^ and denoised with a spatially adaptive filter intended to process images with spatially varying levels of noise^62^. Then, symmetric group-wise normalization was used to derive an anatomical template unbiased with respect to shape and appearance^63^, as implemented in antsMultivariateTemplateConstruction2 script, part of Advanced Normalization Tools (ANTs^64^). Finally, the segmented brain image was obtained with the Brain Extraction Tool (BET^65^), part of FMRIB’s Software Libraries (FSL^66^ v5.0.9). After tissue segmentation, performed with FSL’s Automated Segmentation Tool (FAST^67^ v4), a combined mask of non-grey matter tissue was created.

### Functional data pre-processing

Data pre-processing was performed with FSL v5.0.9. Specifically, the first 5 volumes of each functional series were discarded to avoid initial signal instability. Then, datasets underwent slice-timing correction and motion correction taking the first volume as reference. This reference volume was also used to determine the rigid-body transformation to the corresponding anatomical image. The rigid-body transformation was combined with a non-linear transformation to the group template. The functional images were warped to the prairie vole brain template and resampled to a final resolution of 0.4 × 0.4 × 0.4 mm^3^. Once in the template’s space, the first 5 eigen-vectors (time-series) within the combined non-grey matter mask were obtained to further discard physiological confounds^68^. These eigen-vectors and the 6 motion parameters (3 rotations, 3 displacements) were regressed out from each subject’s functional series. Finally, datasets were band-pass filtered to retain frequencies between 0.01 and 0.1 Hz^69^, and smoothed with a gaussian kernel with a full width at half maximum of 0.8 mm.

### Independent component analysis

To explore the large-scale functional brain networks in prairie voles, group independent component analyses (gICA) were performed on the pre-processed images using FSL’s melodic^1^. The number of components was set to 10 and 30, as these numbers of networks have been previously explored in humans^1,2^ and animal models^16,18,21,37,60^. The gICA maps were scaled to Z-scores and thresholded voxelwise at Z ≥ 2.3 based on a Gaussian/Gamma mixture model and an alternative hypothesis testing approach to identify connected voxels over background voxels^1,70^. The gICA maps were visually inspected and labeled based on their anatomical distribution and location of their maximal regions, visually guided with the Paxinos’ Brain Atlas^71^.

### Data availability

The datasets generated and/or analyzed during the current study are available from the corresponding author.

## Results

### Brain template

**Figure 1:**
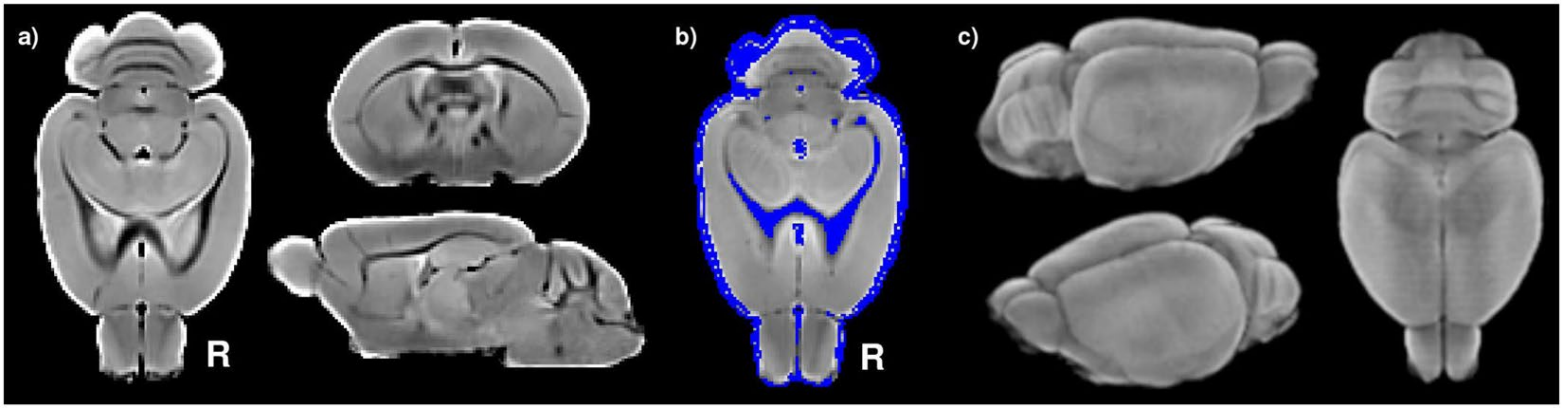
Prairie vole brain template unbiased for shape and intensity^63^. (**a**) axial, coronal and sagittal slices of the isotropic brain template (0.125 mm per voxel side). (**b**) Non-grey matter binary mask (blue), including most of the WM and CSF, overlayed on the brain template. (**c**) 3D-render of the prairie vole brain template.

Magnetic resonance imaging of seven prairie voles were used to create a prairie vole brain template. The anatomical template showed good contrast between grey matter, white matter and cerebrospinal fluid, while brain extraction preserved the gross anatomy including the olfactory bulbs, cerebellum and brainstem (Fig. 1). Tissue segmentation allowed the creation of a binary mask of non-grey matter tissue, including white matter (WM), cerebrospinal fluid (CSF), large vessels and arteries (Fig. 1b), later used to regress out confounding physiological signals (see Methods).

### Independent component analysis

**Figure 2:**
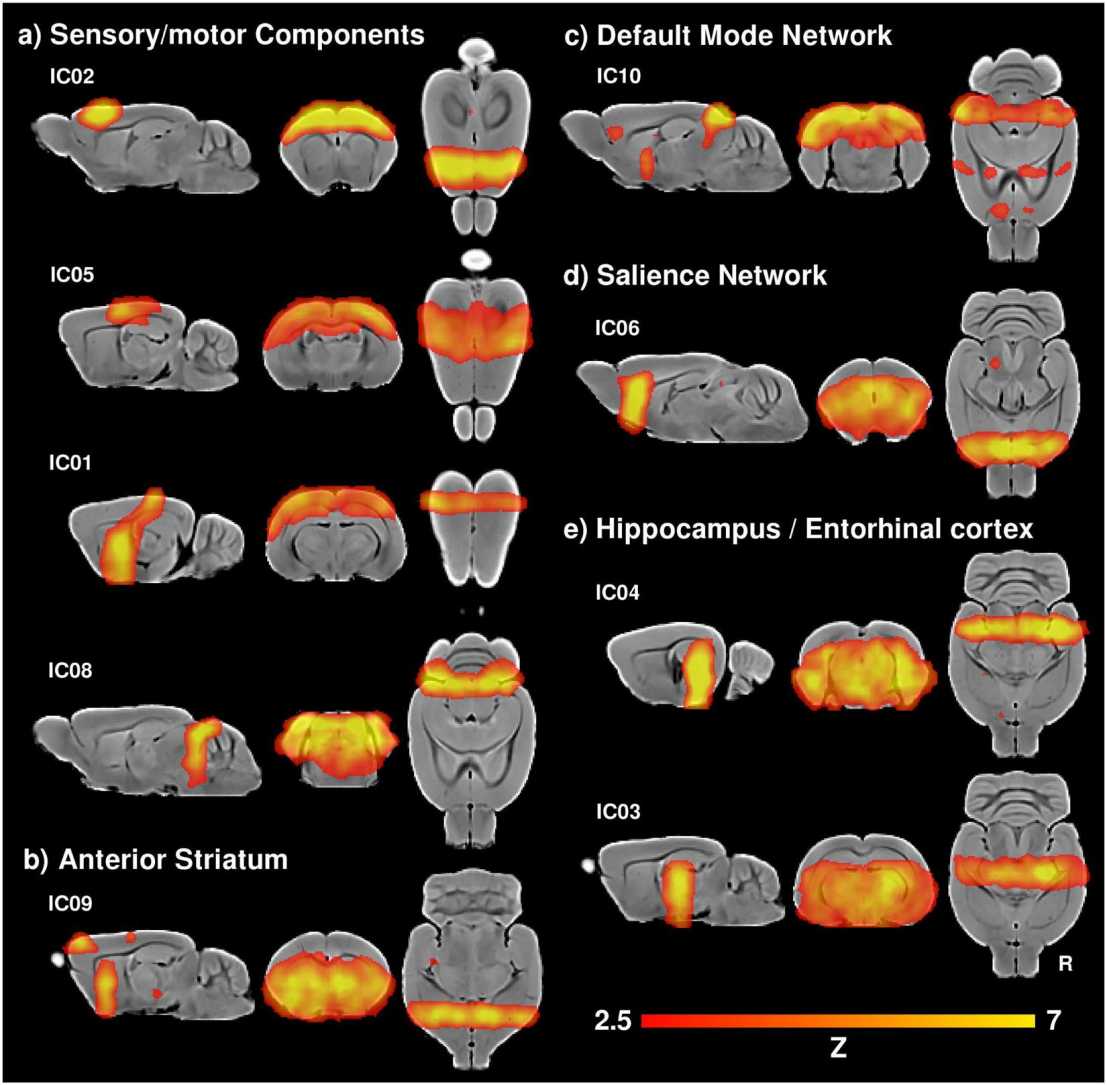
Ten components gICA. (**a**) Sensory/motor components, including motor cortex (IC02), parietal cortex (IC01, IC05), colliculi (IC08). (**b**) Striatum and frontal association cortex. (**c**) Default mode network. (**d**) Salience network. (**e**) Hippocampus and entorhinal cortex (IC03, IC04).

**Figure 3:**
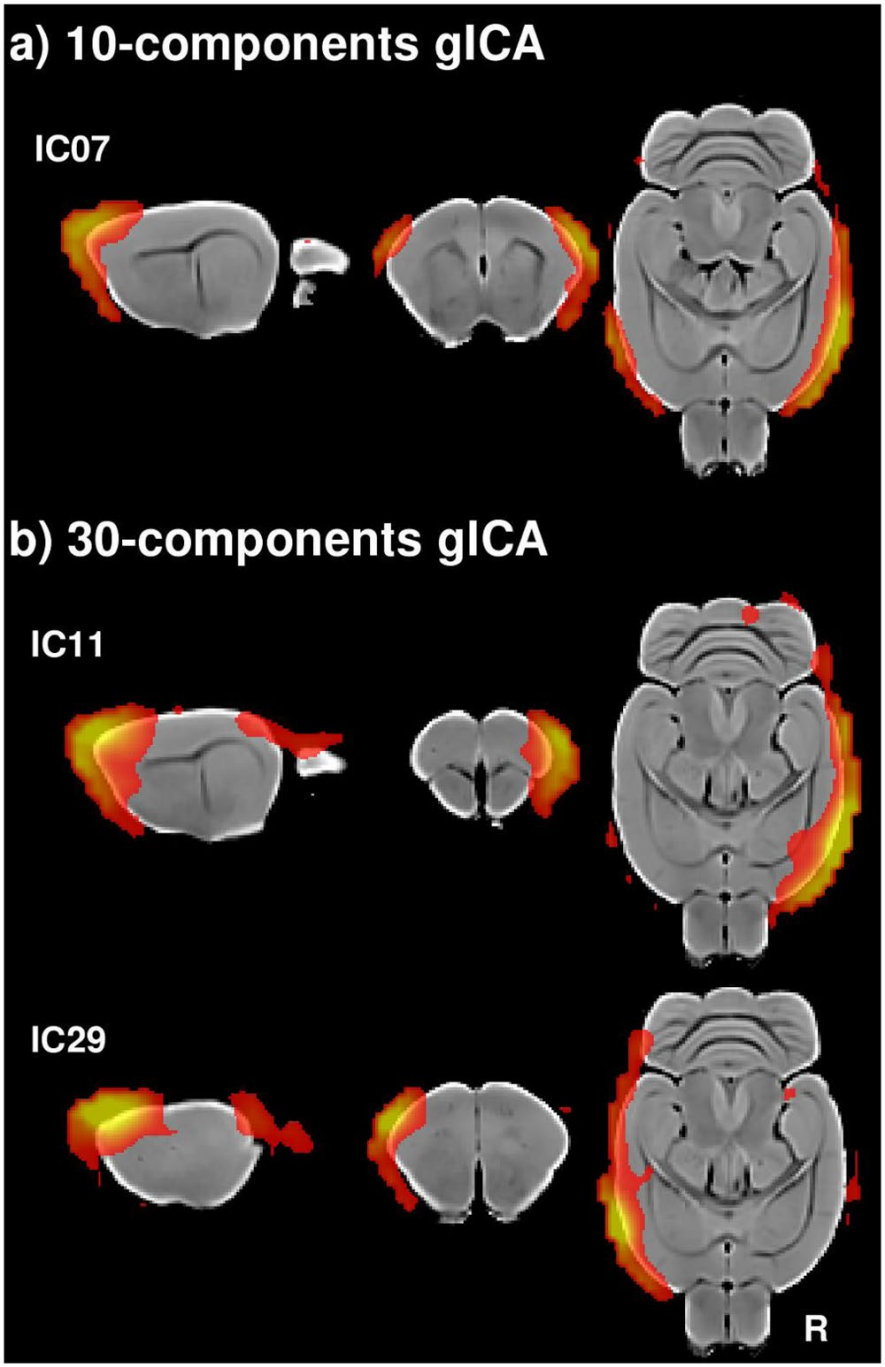
Artifactual components. (**a**) Ten components gICA. (**b**) Thirty components gICA.

The ten-components gICA revealed four components associated with motor and sensory cortices, putative default-mode and salience networks, components centred at the striatum, ventral hippocampi and thalamus, and one non-brain/artifact component (Figs 2 and 3). The identification of the components was based on their spatial distribution and location of their maximal regions, visually guided with the Paxinos’ Brain Atlas^71^. Specifically, three of the four sensory/motor components shown in Fig. 2a revealed large highly symmetric connectivity patterns, mainly covering the motor and somatosensory cortices (IC01, IC02, IC05), but also including connectivity with the striatum and the olfactory bulbs.

The other component labeled as sensory/motor (IC08) also showed a highly symmetric pattern, but showed maximum values at the colliculi and also included bilateral clusters in the caudal occipital lobe (visual cortex). Another component revealed symmetric bilateral functional connectivity of the anterior striatum and the frontal association cortex (IC09; Fig. 2b). The component labeled as the default mode network (IC10) mainly included the retrosplenial (posterior cingulate), occipital (visual), medial orbitofrontal and anterior cingulate cortices, as well as the hippocampus and the striatum (Fig. 2c). The component labeled as the salience network showed local maximum values in the anterior cingulate and the insular region bilaterally (IC06; Fig. 2d). Two components covered the hippocampus and entorhinal cortex, one also including the bilateral thalamus (IC03, IC04; Fig. 2e), these regions have been also related to the default mode network^72^. Finally, one non-brain component was identified, showing large clusters with maximal values out of brain (Fig. 3a). This component seems to reflect residual influence of the CSF signal.

**Figure 4:**
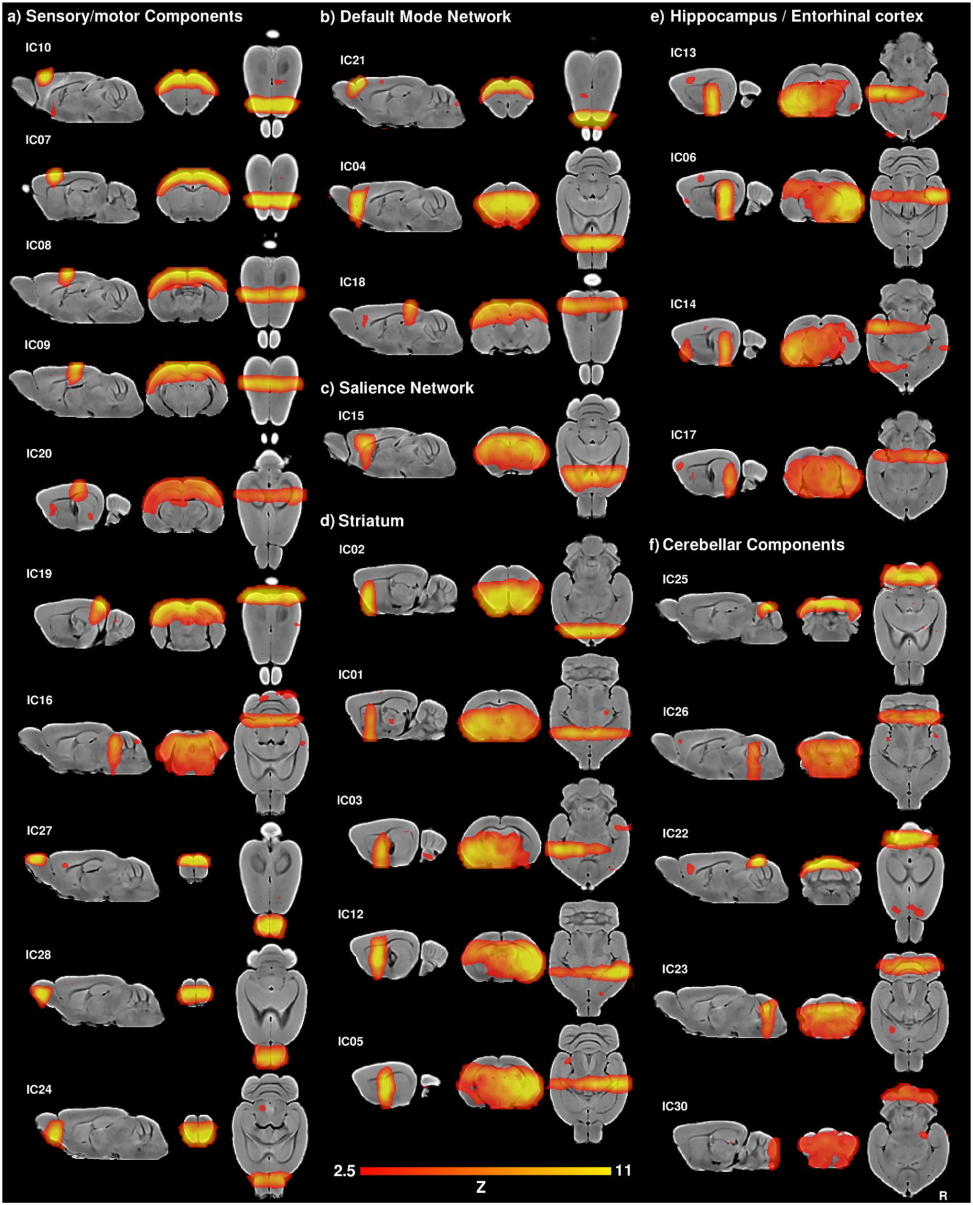
Thirty components gICA. (**a**) Sensory/motor components, including motor and somatosensory (IC07- IC10, IC20), occipital (visual) cortex (IC19), colliculi (IC16) and olfactory bulbs (IC24, IC27, IC28). (**b**) Default mode network, including the frontal association cortex (IC21), orbitofrontal cortex (IC04), retrosplenial and parietal cortex (IC18). (**c**) Salience network, including anterior cingulate cortex and insulae (IC15). (**d**) Striatum. (**e**) Hippocampus and entorhinal cortex. (**f**) Cerebellar components.

The thirty-components gICA revealed some of the components identified in the lower dimension analysis, however it also showed regions with no significant connectivity in the previous analysis. Specifically, several motor and sensory components were evident, putative default mode and salience networks were also identified, although default mode network was subdivided in at least three components, and as in the previous analysis, some components were anchored in the striatum, within the hippocampus or entorhinal cortex (Fig. 4). In addition, three components covered the olfactory bulbs (Fig. 4a) and five cerebellar components were further identified (Fig. 4f). Finally, two components were identified as non-brain related or artifactual components (Fig. 3b).

Among the sensory-related components, six components showed similar patterns as in the lower dimension gICA, revealing large highly symmetric connectivity patterns, mainly covering the motor, somatosensory and visual cortices (IC07-IC10, IC19, IC20; Fig. 4a). Also similar to the lower dimension gICA, one component showed a highly symmetric pattern with maximum values at the colliculi (IC16; Fig. 4a). In contrast to the lower dimension gICA, the olfactory bulb displayed strong functional connectivity patterns (Fig. 4a), being segregated into three components: anterior dorsal (IC27), anterior ventral (IC28) and posterior ventral (IC24).

Three components resembled the default mode network, two anterior and one posterior (Fig. 4b). The anterior components included the frontal association (IC21) and the orbitofrontal (IC04) cortices, while the posterior component included the retrosplenial and parietal association cortices (IC18). The putative salience network, consistent to that identified in the lower dimension analysis, is also similar to that previously identified in the mouse brain^15,22^, which included maximal values in the anterior cingulate and the insular regions (IC15; Fig. 4c).

Five components showed maximum values within the striatum and adjacent cortices (Fig. 4d). Of these striaitum-centred components, one showed maximal values in the anterior ventral striatum, bilaterally, while the other four components mainly showed unilateral connectivity patterns, with two components showing maximal values in the left hemisphere (IC01, IC03) and other two in the right hemisphere (IC05, IC12). Other four components showed mainly unilateral connectivity patterns within the hippocampus and the entorhinal cortex, two in each hemisphere (Fig. 4e).

In contrast to the lower dimension gICA, several components showed functional connectivity patterns anchored in the cerebellum. Specifically, five components showed bilateral distribution mainly within the cerebellum, but also showed some connectivity with the frontal cortex, striatum and hippocampus (Fig. 4f). Finally, two components from the high dimension gICA were labeled as non-brain components or artifacts (Fig. 3b), showing large clusters with maximum values out of the brain, laterally (IC11, IC29), similar to the artifactual component identified in the ten-components gICA (IC07; Fig. 3a).

## Discussion

We have identified motor, sensory and high order resting state brain networks in the prairie vole, an extraordinary animal model to study the neurobiology of complex social behavior^40,73^. The identified resting state networks, including the default mode and salience networks, add to those identified in other rodents^15,17–20^, ferrets^60^, non-human primates^24,25,28,74,75^ and humans^1,2,4^. These findings support the use of resting state fMRI to characterize the functional organization of the prairie vole brain, with potential applications in the basic and translational research of social-related behavior.

Several similarities are evident in the components identified by the low- and high-dimension gICAs. First, both gICA approaches revealed highly symmetric connectivity patterns anchored in the motor and somatosensory cortices (Figs 2a and 4a). Specifically, the components IC02 and IC10 of the low- and high-dimension gICA, respectively, mainly cover primary and secondary motor cortices, while the components IC01 and IC05 in the low-dimension gICA and the IC07 – IC09 of the high-dimension gICA, seemed to cover both somatosensory and motor cortices. The overlap of somatosensory and motor cortices within the same resting state component is not surprising given the cortical sensorimotor integration identified in both rodents and humans^76–78^. Indeed, humans usually exhibit the somatosensory and motor cortices within the same component, referred as the sensorimotor network^1,2,4^. In addition, both analyses also identified a strikingly similar component driven by the colliculi and a component including the caudal occipital lobes (visual cortex) and superior colliculi. These results evidence robust sensory- and motor-related resting state networks, similar to those identified in the mouse and rat brains^16–18,20–22,72^.

Second, putative default-mode and salience network components were identified with both the low- and high-dimension analyses. Specifically, the low-dimension analysis identified a single default mode network component including the retrosplenial, occipital (visual) and medial orbitofrontal cortex, as well as the posterior striatum and hippocampus (Fig. 2b). In contrast, the high-dimension analysis evidenced three components related to the default mode network, two anterior and one posterior (Fig. 4b). The anterior components included the frontal association (IC21) and the orbitofrontal (IC04) cortices, while the posterior component included the retrosplenial and parietal association cortices (IC18). Previous studies focusing on the resting state functional MRI of the rodent brain have also identified similar connectivity maps as a putative default mode network. In particular, a low-dimension gICA analysis (5 components) identified a single component including the retrosplenial cortex as well as the anterior cingulate and orbitofrontal cortices among other structures in the mouse brain^22^. However, when exploring higher dimension gICA several studies have shown the default mode network segregated in at least anterior and posterior components in mice and rats^15,22,23,72^. Other animal studies have similarly shown anterior and posterior components in ferrets^60^ and non-human primates^24,27,28,75,79,80^. This segregation pattern is consistent with the modular nature of the default mode network in the human brain, with the medial prefrontal and posterior cingulate cortices identified as two main hubs for brain functional connectivity^81–83^.

Third, subcortical components including the striatum and hippocampus were evident in both gICA analyses. However, the lower dimension gICA identified bilateral subcortical components while the higher dimension analysis showed higher number of components that were also segregated into unilateral patterns (Figs 2 and 4). These results are consistent with previous studies using ICA that showed bilateral and unilateral components within the hippocampus and striatum in the rodent brain^17,18,20,72^.

In addition to the consistent networks identified in both analyses, the higher dimension gICA allowed the identification of components that were not evident in the lower dimension analysis. Specifically, three connectivity maps within the olfactory bulbs and five cerebellum-specific components were evident only in the higher dimension gICA. These regions may have been part of the lower dimension components but at a lower significance level and their connectivity patterns may reflect intrinsic functional segregation within olfactory bulbs and cerebellum. The division of the olfactory bulbs in three components, a posterior ventral segment and two rostral components, one dorsal and one ventral, might be related to the functional organization of the olfactory bulbs. Indeed, some studies have described that glomeruli, the odorant receptors in the olfactory bulbs, organize into clusters that are sensitive to a similar combination of molecular features, and at least two sets of clusters have been defined, one in the dorsal portion and the other in the lateral posterior portion of the olfactory bulbs^84^, which seem consistent with the general distribution of the components here identified. However, more research is needed to elucidate the functional role of these large scale segregation patterns.

Altogether, the components identified in the low- and high-dimension gICA are evidence of consistent resting state networks with modular and hierarchical organization similar to those identified in other species and in the human brain. However, further investigation is needed in order to explore the functional relevance of such connectivity patterns and their potential use as biomarkers for health, neurobiological function and pathology in the prairie vole.

Similar to our study, detection of the default mode network in rodents and ferrets has been based on the anatomical distribution of the functional connectivity maps, including the medial prefrontal and retrosplenial cortices. A proper definition of the default mode network would imply the detection of decreased levels of connectivity while performing externally orientated tasks^30,31,85^, which has been shown in non-human primates^24,27,79^, but practically unexplored in rodents given the complexities of implementing task-based functional imaging. However, there is some evidence that supports the functional meaning of the rodent default mode network. Using the correlation of amperometric oxygen readouts^86,87^ as a measure of functional connectivity in rats performing sustained periods of instrumental response alternated with spontaneous behavior, a recent study showed task-induced modulation within the default mode network (between the prelimbic and retrosplenial cortices), but not in a sensory-motor lateral cortical network^88^. These results within network task-based modulation are consistent with human findings^30,31,85^ supporting the existence of at least a precursor of the default mode network in the rodent brain.

Although human studies have attributed the relevance of the default mode network to high order functions related to human social behavior such as self-consciousness^89^ and theory of mind^90^, some recent results suggest the relevance of the integrity of the default mode network in rodents for preserved social-related behavior. It has been shown that rats exposed to chronic stress, which results in behavioral alterations related to anxiety and depression, exhibit increased functional connectivity within the default mode network^91^. Similarly, rsfMRI has already shown alterations of this network in a series of rodent models of autism^38,92^ and depression induced by psychosocial stress^36^. These results suggest a plausible link between the putative default mode network and social behavior in the rodent brain, and warrant further investigation in the prairie vole, a species that displays more complex social behaviors than mice and rats.

It is clearly established that modulation of the reward system in the prairie vole is fundamental for social bonding. It has been shown for example that arginine vasopressin (AVP), oxytocin (OT) and dopamine (DA) facilitate pair bonding in the vole^44,93–95^. There is also data indicating that in male prairie voles, one ejaculation and mating in conditions that lead to pair bonding, involve the opioid-mediated reward^96^. In addition, the density of dopamine receptors significantly increases in the nucleus accumbens after two weeks of maintained partner preference^97^, indicating that plastic changes in the reward system are associated with social bonding. Furthermore, using optogenetics and electrophysiology, it has been recently shown that modulation of corticostriatal functional connectivity enhances affiliative behavior to a partner^98^. In humans, neuroimaging studies focusing on romantic and parental love have documented the relevant role of brain networks associated to reward, motivation and emotion^99,100^. It has been shown for example, that romantic love increases the resting state functional connectivity of reward, motivation and memory networks^99^. Future studies will need to test if prairie voles show any differences in the resting state functional connectivity before and after pair bonding, particularly between frontal cortices and the striatum.

Several neuroimaging studies in psychiatric disorders involving abnormal social behavior have identified altered brain functional connectivity in the human brain. Among them, autism spectrum disorders (ASD) have been related to a myriad of alterations in resting state functional connectivity^101^. Neuroimaging research in animal models may help to identify the neural basis of such findings^102^. Indeed, a recent study exploring a genetically modified mouse model (*cntnap2-*null), recreating some of the social impairments characteristic of autism, demonstrated decreased long-range and local resting state functional connectivity in prefrontal and midline brain^38^. In particular, decreased social investigation^38^ was associated with altered connectivity of the default mode network. Given that the prairie vole is a natural model to explore human-like social behavior, functional neuroimaging of the prairie vole should greatly expand our knowledge of the neurobiology of the human findings in ASD. In particular, prairie voles and humans show similar genetic variations of the genes associated with the distribution of vasopressin 1a receptor (avpr1a)^103,104^ and the oxytocin receptor (Oxtr)^45^, which in humans may contribute to the altered social behavior identified in ASD^105–108^. Future research should explore the similarity of the resting state functional connectivity in both species and its relation to such genetic variations and associated social behavior.

Social anxiety disorder, a chronic psychiatric disorder that leads to avoidance of social situations, has been associated with decreased functional connectivity between the nucleus accumbens and prefrontal cortex^109^. This evidence suggests that similar but opposed mechanisms may mediate social bonding and social avoidance, and that rsfMRI provides a means to identify their underlying brain circuits. Social isolation in prairie voles impaires neurogenesis^110^, produces alterations in neuroendocrine function^111^ and induces behavioral changes that resemble anxiety and mood disorders^112,113^. The question arises if in the prairie vole we could detect changes in the resting state connectivity as those identified in humans with anxiety and mood disorders^114–118^. Future studies will also examine the effect on resting state networks of early-life neglect and genetic variation in the Oxtr and avpr1a, as well as gene by environment interactions, known to influence social behavior^45,53,103^.

A potential limitation of this study is the exploration of functional connectivity in anesthetized animals, which may limit brain functional connectivity. However, we used a minimal dose of isoflurane (~1%). It has been shown that higher doses of isoflurane disrupt bilateral cortical connectivity and cortical-subcortical connectivity in mice^16^. However, the dose used here allowed the identification of both bilateral and cortico-subcortical connectivity. Although functional neuroimaging in the awake prairie vole is possible, the acclimation process doesn’t avoid significant stress and movement artifacts and may even increase the risk of physical harm to the voles^59^. Although this procedure is extremely valuable when comparing task-related brain activation, the induced stress would limit the interpretation of the results as stress significantly affects resting state functional connectivity^119^ and promotes behavioral changes associated with anxiety and depression^73,112^. In the present study we have only explored male prairie voles, future studies should also evaluate females and even compare if any sex differences are evident in the resting state networks, since many of the neurobiological mechanisms of pair bonding are sex specific^39,40^.

Our results confirm the presence of spontaneous homotopic inter-hemispheric resting state connectivity networks in the prairie vole brain, highly consistent with those identified in rodents and humans, providing the basis and support for the use of rsfMRI for basic and translational research to understand the complex social behaviors expressed in this species. It’s expected that rsfMRI will help to close the gap between the properties of the large scale functional organization and the underlying biomolecular and cellular mechanisms of several aspects of social cognition, including social attachment, social buffering and nurturing behavior, with important implications for the study of normal development, addiction and neuropsychiatric disorders.
